# The Detection of Neutrophil Activation by Automated Blood Cell Counter in Sepsis

**DOI:** 10.14789/jmj.JMJ23-0044-P

**Published:** 2024-03-18

**Authors:** JULIE HELMS, FERHAT MEZIANI, LAURENT MAUVIEUX, TOSHIAKI IBA

**Affiliations:** 1 Strasbourg University (UNISTRA), Strasbourg, France; 1 Strasbourg University (UNISTRA), Strasbourg, France; 2 Strasbourg University Hospital, Medical Intensive Care Unit - NHC, Strasbourg, France; 2 Strasbourg University Hospital, Medical Intensive Care Unit - NHC, Strasbourg, France; 3 INSERM (French National Institute of Health and Medical Research), UMR 1260, Regenerative Nanomedicine (RNM), FMTS, Strasbourg, France; 3 INSERM (French National Institute of Health and Medical Research), UMR 1260, Regenerative Nanomedicine (RNM), FMTS, Strasbourg, France; 4 Laboratory of Hematology and Hemostasis, Strasbourg University Hospital, Strasbourg, France; 4 Laboratory of Hematology and Hemostasis, Strasbourg University Hospital, Strasbourg, France; 5 UPR3572 CNRS I2CT -Immunology, Immunopathology and Therapeutic Chemistry, Strasbourg, France; 5 UPR3572 CNRS I2CT -Immunology, Immunopathology and Therapeutic Chemistry, Strasbourg, France; 6 Department of Emergency and Disaster Medicine, Juntendo University Graduate School of Medicine, Tokyo, Japan; 6 Department of Emergency and Disaster Medicine, Juntendo University Graduate School of Medicine, Tokyo, Japan

**Keywords:** sepsis, neutrophil, cell count, cell death, neutrophil extracellular traps

## Abstract

Neutrophils serve as the frontline defenders in the host's response to infections. However, the available methods for assessing the activated status of neutrophils are still limited. The immature cells that appear during sepsis are large with complex cytoplasmic components and rich nucleic acids, making them diagnosable by cell population data analysis using the automated cell counter. The changes are expressed as increased forward scattered light, side fluorescence light, and side fluorescence distribution width. Additionally, changes in side fluorescence light may indicate the neutrophil extracellular trap formation and can be useful for the diagnosis of sepsis-associated disseminated intravascular coagulation.

## Introduction

**Figure 1:**
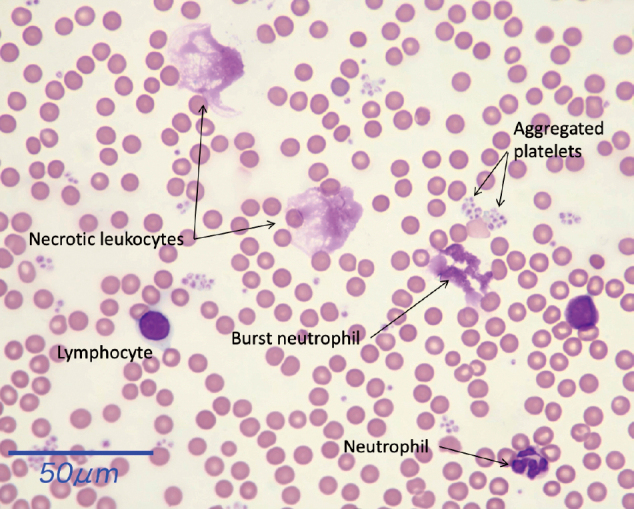
Peripheral blood smear findings in sepsis Sepsis was induced by *E.coli* injection to the rats. The blood sample was collected, and the blood smear was fixed with methanol and stained with May-Grunwald Giemsa. Some neutrophils were enlarged, and the nuclear contents were expelled outside the cells.

Neutrophil is the most abundant and fast-reacting leukocytes in sepsis. Recent research elucidated the critical roles of neutrophils in the frontline of the host defense against infection. They demonstrate bacteriocidal activity even after cell death. Other than apoptotic cell death, proinflammatory cell death such as necrosis, pyroptosis, and ferroptosis increased during sepsis, and the blood smear findings revealed disrupted nuclear membranes with the dispersion of nuclear contents and the presence of burst neutrophils^1)^ (Figure 1). In addition, turnover of the cell cycle is increased, and an accelerated turnover is partially detected by the increased cell counts and the presence of immature neutrophils namely, band neutrophils. However, other parameters are not easily assessable.

## NE-WY and NE-SFL, markers of bacterial sepsis?

**Figure 2:**
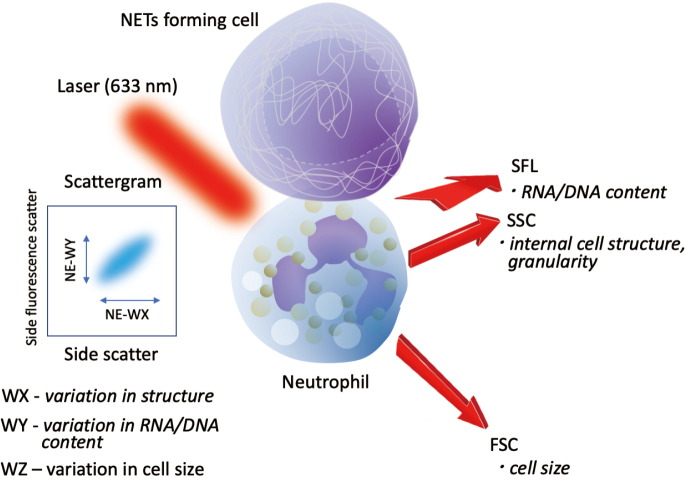
The mechanism of cell population data analysis Cell population data were obtained with an automated hematological analyzer. The analyzer allows the measurement of neutrophil fluorescence by impedance and by fluorescence flow cytometry at 633 nm. The analysis included the labeling of neutrophils with fluorescent dyes. Signal fluorescence intensity was used to measure the RNA/DNA content.

Park et al.^2)^ reported the usefulness of the specific automated blood cell counter for dividing the neutrophils into subtypes depending on their phenotypes. Popular cell counters (cell analyzer), such as Sysmex XN20^®^ analyzer (Sysmex Corporation, Kobe, Japan) and DxH800^®^ (Beckman Coulter Inc., Miami, FL, USA) are able to analyze the morphological characteristics of cells and provide information about various cell population data (CPD) that reflect the detailed status of neutrophil activation. The neutrophil parameters obtained by representative cell analyzer Sysmex XN-20 were as follows: forward scattered light (NE-FSC), side scattered light (NE-SSC), side fluorescence light (NE-SFL), and the variances of the above indicators. Those are side scattering light distribution width (NE-WX), side fluorescence distribution width (NE-WY), and forward scattering light distribution width (NE-WZ) (Figure 2). Among them, variations in RNA/DNA contents represented by NE-WY and NE-SFL were reported to be significantly higher in patients with sepsis compared to the healthy controls^2-4)^. In a population of patients at the onset of fever, NE-SFL, NE-WY, NE-WZ, and Monocytes-WZ parameters reached the highest AUC scores for predicting sepsis^5)^. Furthermore, unsupervised K-means clustering in the sepsis group separated patients with high procalcitonin from the others. Since immature neutrophils are rich in nucleic acids, the increase in immature cells can explain the high levels of NE-WY and NE-SFL. Narumi et al.^6)^ reported that there were no significant differences in the NE-WY or NE-SFL among patients with sepsis, bacteremia, and focal infection. However, they showed that NE-WY and NE-SFL showed a very high differentiation ability for sepsis, and NE-WY, NE-SFL, and NE-FSC were independent predictors of sepsis^7)^. These findings reflected the blood smear findings of increased immature cells, complex internal structure, and specific morphology, such as the presence of toxic granules and vacuolization. Notably, NE-FSC was significantly lower in patients with sepsis in their study. Low NE-FSC indicates the decrease in mean cell size which was ambivalent to the increase of immature neutrophils. Since apoptotic neutrophils are increased in sepsis, decreased NE-FSC with increased NE-WZ reflect the mixture of apoptotic and immature neutrophils. Although the usefulness of CPD analysis for the diagnosis of sepsis warranted further study, it is noteworthy that the above parameters are easily and quickly calculated from the data of a complete blood count without additional cost.

## NE-SFL, a marker of sepsis-induced disseminated intravascular coagulation?

Disseminated intravascular coagulation (DIC) is a critical complication of sepsis, and disease severity is known to increase considerably when patients are complicated by DIC. A multicenter study revealed the prevalence of DIC was 45.7% in sepsis due to acute respiratory distress syndrome, with a mortality rate reaching 40.7%^8)^. These data were confirmed in two prospective multicenter cohorts of septic shock patients, in which 43 and 36% of the patients - respectively - developed DIC^9, 10)^. DIC was strongly associated with septic shock severity, sequential organ failure assessment (SOFA), and mortality (45.2% in DIC group versus 28.3% in non-DIC, p<0.001). Neutrophil activation plays a pivotal role in the development of DIC by upregulating thromboinflammation in the vasculature^11)^. NETosis is a type of cell death with releasing neutrophil extracellular traps (NETs) and is involved in the development of immunothrombus and DIC^12)^.

In 100 septic shock patients - including 35 DIC - Stiel et al.^13)^ reported that NE-SFL was significantly higher in patients with DIC compared to non-DIC patients: 66.6 (59.3-80.4) versus 50.0 (46.6-56.2) (p<0.01). With a cut-off at 57.3 arbitrary units, the area under the ROC curve was 0.882 (p<0.0001) for early DIC diagnosis, with a sensibility is 90.91% and a specificity of 80.60%. Interestingly, NE-SFL values were increased *in vitro* in a range identical to that observed in septic patients in ionomycin-induced NETosis in blood samples from healthy subjects, while NE-FSC and NE-SSC were not significantly different.

Delabranche et al.^14)^ confirmed the potential link between NETosis and DNA decompaction expressed by NE-SFL, by showing that indirect markers of NETosis (nucleosomes and DNA-myeloperoxidase) were significantly increased in DIC patients and that NE-SFL, NETs, and elevated nucleosome concentrations were all correlated to DIC (p<0.05).

Finally, Stiel et al.^15)^ detected circulating NETs forming cells using an immunofluorescent staining technique in the peripheral blood obtained from patients with sepsis and DIC. At the same time, they reported that the chromatin decompaction during the pathway of NETosis could be detected by CPD analysis.

The appearance of NETosis is characterized by the loss of intracellular membranes before the integrity of the plasma membrane is compromised. As a result, decondensed chromatin spreads in the cytoplasm, and the cells expand with the damage to the cellular membrane^16)^. Therefore, automated cell analyzers can capture the increased variation in cell size expressed by large NE-WZ and increased cell size expressed by large NE-FSC^2)^.

In summary, CPD using an automated cell analyzer is a low-cost, routinely available, and rapid measure to evaluate neutrophil activation. Neutrophil activation is represented by the presence of large and chromatin-rich immature neutrophils and reactive neutrophils with toxic granules and vacuolization, which can be detected by CPD analysis. Although it is unknown whether NETosis or other types of cell death can be accurately detected by this measure, the CPD assessment is promising for early detection of sepsis and sepsis-associated DIC.

## Funding

No funding was received.

## Author contributions

JH and TI wrote and reviewed the manuscript. FM and LM revised the manuscript. All authors read and approved the final manuscript.

## Conflicts of interest statement

The authors declare that they have not conflict of interest.
